# Hemostatic efficacy and safety of the hemostatic powder UI-EWD in patients with lower gastrointestinal bleeding

**DOI:** 10.1186/s12876-022-02247-4

**Published:** 2022-04-07

**Authors:** Boram Cha, Donghyun Lee, Jongbeom Shin, Jin-Seok Park, Gye-suk Kwon, Hyungkil Kim

**Affiliations:** Digestive Disease Center, Department of Internal Medicine, Inha University Hospital, Inha University School of Medicine, 27 Inhang-ro, Jung-gu, Incheon, 22332 South Korea

**Keywords:** Lower gastrointestinal bleeding, Hemostatic UI-EWD powder, Re-bleeding, Safety

## Abstract

**Background and aims:**

Acute lower gastrointestinal bleeding (LGIB) is a common cause of emergency hospitalization and may require readmission for re-bleeding. Recently, a novel endoscopic hemostatic powder (UI-EWD/Nexpowder™, Nextbiomedical, Incheon, South Korea) was developed and applied for the control of LGIB. The aim of this study was to evaluate the hemostatic efficacy and long-term safety of UI-EWD in LGIB.

**Patients and methods:**

We conducted a retrospective cohort study of LGIB at a single tertiary center in south Korea. One hundred and sixty-seven consecutive patients with LGIB who were initially successful in endoscopic hemostasis were included and divided into the conventional treatment group (n = 112) and the UI-EWD therapy group (n = 55; 38 patients with conventional treatment and 17 patients with UI-EWD alone). The success rate of hemostasis, adverse events related to UI-EWD, and re-bleeding rate were evaluated.

**Results:**

The incidence of endoscopic hemostasis applied to the hepatic flexure (7.3% vs. 0%, *p* = 0.011) and larger than 4 cm (25.5% vs. 8.0%, *p* = 0.002) were significantly higher in the UI-EWD group than in the conventional therapy group. The cumulative rebleeding rate within 28 days in the UI-EWD group was 5.5% (3/55), which was significantly lower than that in the conventional treatment group (17.0% [19/112]; *p* = 0.039). No UI-EWD-related adverse events were recorded.

**Conclusion:**

Based on our results, application of UI-EWD in LGIB showed promising results for the prevention of re-bleeding, especially in locations where it is difficult to approach or cases with more bleeding. There were no significant complications, such as perforation or embolism. In particular, UI-EWD should be considered first for anatomical or technical impediments to endoscopic access in LGIB.

## Introduction

Low gastrointestinal bleeding (LGIB) accounts for 20–25% of all gastrointestinal (GI) bleeding episodes [1, 2]. Although LGIB is relatively less common than upper GI bleeding, it remains a frequent cause of hospitalization, with a mortality rate of up to 20% [3, 4]. Despite high success in the initial hemostasis of LGIB, the re-bleeding rate is still high, up to 16.8% [5]. Moreover, limitations remain despite the recent development of endoscopic hemostasis technology, including cautery, thermocoagulation, clip, injection therapy, and argon plasma coagulation [6]. Considering the high risk of perforation due to the thinner wall of the colon compared to the stomach, endoscopists tend to be limited in their endoscopic hemostasis, especially in LGIB. Indeed, hemoclips are not suitable for diffuse bleeding lesions and can cause mechanical mucosal damage by the device. Although thermocoagulation is suitable for large bleeding surfaces, endoscopists need to consider coagulation syndrome and delayed perforation [1, 7].

To compensate for these limitations, hemostatic powders such as Hemospray (also known as TC-325) (Cook Medical, Winston-Salem, NC, USA) [8, 9], EndoClot (AMP; EndoClot Plus Inc., Santa Clara, CA, USA) [10, 11], and ABS (Ankaferd Health Products Ltd., Istanbul, Turkey) [12] have been developed as alternative non-contact and non-traumatic endoscopic tools, especially for diffuse and large bleeding lesions, with excellent initial hemostatic rates of up to 98% in upper GI bleeding [13, 14]. Moreover, previous reports have also shown the high hemostatic efficacy of hemostatic powders in LGIB [15–17].

Although the effects of lowering the rebleeding rate of hemostatic powders on upper and lower gastrointestinal bleeding have been reported, even with the addition of hemostatic powders, higher rebleeding rates of up to 13% in LGIB powders remains a challenge for endoscopists [16]. A highly adhesive hemostatic powder (UI-EWD, Nextbiomedical, Incheon, South Korea), which is a biocompatible natural polymer consisting of aldehyde dextran and succinic acid modified ε-poly, has been newly developed. Recently, good hemostatic efficacy of UI-EWD on refractory upper GI bleeding, non-variceal bleeding, and diffuse tumor bleeding, with low rebleeding rates was reported [18–22]. However, no previous study has examined the hemostatic effects of UI-EWD in patients with LGIB.

We aimed to retrospectively evaluate the hemostatic efficacy and safety of UI-EWD in patients with LGIB.

## Patients and methods

### Study design and study population

**Fig. 1:**
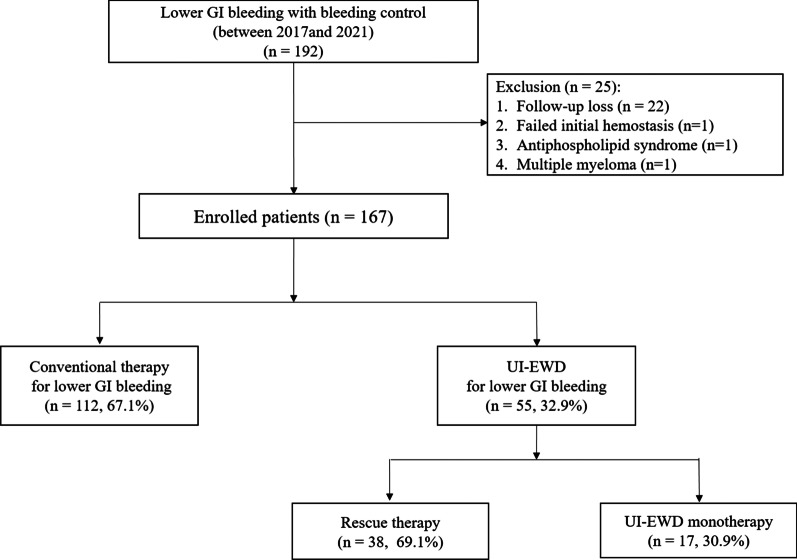
Patient selection flowchart of the study

Patients treated for LGIB between January 2017 and July 2021 at Inha University Hospital were enrolled. The patients were retrospectively selected from established prospective registries based on the following inclusion criteria: (1) age > 18 years at the time of treatment; (2) suspected active LGIB (e.g., hematochezia or melena); and (3) past endoscopic hemostasis. The exclusion criteria were as follows: (1) upper GI bleeding; (2) hematologic disorder; (3) autoimmune disease; (4) pregnancy or suspected pregnancy at the time of treatment; and (5) receipt of treatment by other endoscopic or surgical treatment within 30 days prior to UI-EWD application. Finally, 167 patients met the inclusion and exclusion criteria (Fig. 1). The medical records of the included patients were reviewed. Information on clinical characteristics, bleeding, clinical outcomes (including immediate hemostasis success and re-bleeding rates), and hemostasis-related complications, including UI-EWD-associated adverse events, were collected.

### Endoscopic procedures

**Fig. 2:**
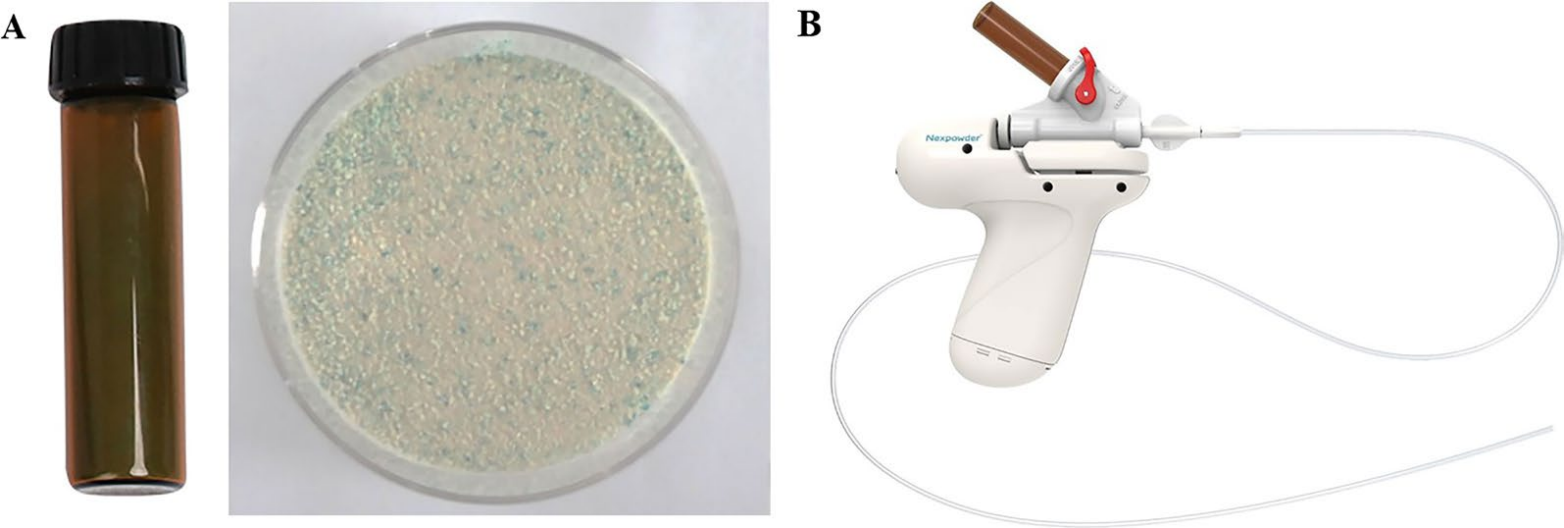
UI-EWD (**A**) and powder delivery devices (**B**)

**Fig. 3:**
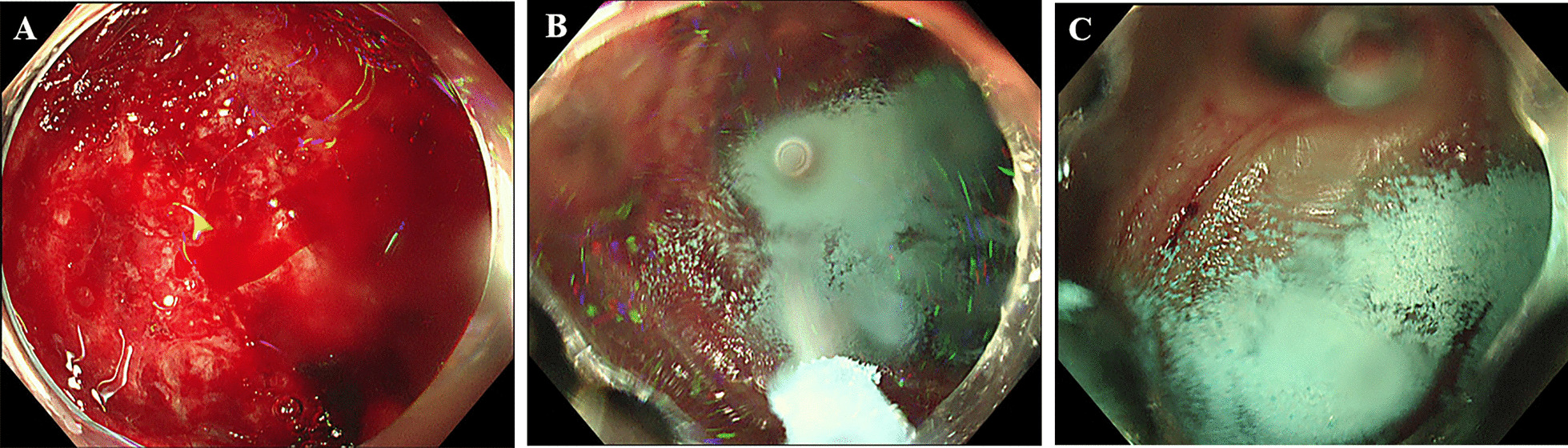
Endoscopic images of UI-EWD application in LGIB. **A** Case of diffuse oozing bleeding due to an ulcer on the hepatic flexure before the application of UI-EWD. **B** UI-EWD monotherapy was decided and applied due to anatomic difficulty in approaching by conventional treatment. **C** Successful hemostasis was confirmed 5 min after the application of the UI-EWD

UI-EWD was applied to LGIB using a conventional endoscope (H290-TL/I, Olympus, Tokyo, Japan) by experienced endoscopists (6–25 years). In the UI-EWD group, the final hemostasis sequence was UI-EWD. UI-EWD was used as monotherapy or rescue therapy when the endoscopist determined the most appropriate time to apply UI-EWD to LGIB. UI-EWD monotherapy was applied when bleeding was minimal and was enough for the single use of UI-EWD or on diffuse oozing bleeding that might not be treated effectively by conventional therapy. Rescue therapy was defined as additional treatment when continuous bleeding was observed, even after conventional treatment. UI-EWD was sprayed onto the surface of the bleeding site using a catheter passed through the powder delivery system (Fig. 2) under direct endoscopic vision until the bleeding lesion was completely covered with the powder (Fig. 3). UI-EWD was applied with a maximum release of 6 g of powder.

The antithrombotic agents were discontinued in patients who were on antithrombotic therapy. The sips of water was started when there was no evidence of bleeding at 24 h after endoscopic hemostasis, and antithrombotic therapy was resumed as soon as possible if there was no evidence of bleeding after sips of water. The time to restart the antithrombotic therapy was within 48 to 72 h after hemostasis.

### Outcome measurements

Successful initial hemostasis was defined as confirmation of hemostasis until 5 min after the endoscopic treatment. If bleeding persisted after 5 min with conventional hemostasis, UI-EWD was applied as a rescue therapy at the endoscopist’s discretion. Immediate hemostasis failure was defined as cases in which additional application of treatment modalities was required after applying UI-EWD. Rebleeding was defined as clinical evidence of bleeding, such as melena or hematemesis, with an associated reduction of 2 g/dL of hemoglobin within 28 days after the initial successful endoscopic hemostasis [23]. When re-bleeding was suspected, further endoscopic evaluation was performed to confirm the actual bleeding status. The patients’ medical records were reviewed to assess the adverse events associated with UI-EWD, such as newly developed symptoms (e.g., abdominal pain, nausea, and vomiting), changes in vital signs, detection of free air on abdominal plain radiography, and laboratory test abnormalities. Moreover, the possibility of embolism, intestinal obstruction, and allergic reactions due to the characteristics of the powder were determined.

### Statistical analysis

The clinical characteristics of the study subjects were expressed as medians (ranges) for continuous variables and numbers (percentages) for categorical variables. The differences between categorical or continuous variables were analyzed using the Mann–Whitney U test, Student’s t-test, chi-square test, or Fisher’s exact test. The overall re-bleeding and cumulative survival rates were estimated using the Kaplan–Meier method. Two-tailed *p*-values < 0.05 were considered statistically significant. Statistical analyses were performed using SPSS v25.0 (SPSS Inc., Chicago, IL, USA).

## Results

### Baseline clinical characteristics of the conventional group and the UI-EWD therapy group

Between March 2017 and July 2021, 192 patients were treated for LGIB. In one patient, treatment was not possible because of excessive bleeding, and 25 patients were excluded based on the exclusion criteria. Finally, 167 patients were enrolled in the study. The baseline clinical characteristics of the conventional treatment and UI-EWD therapy patients are shown in Table 1. Conventional therapy for lower GI bleeding was performed in 112 patients, and UI-EWD was applied during endoscopic treatment in 55 patients. UI-EWD was used as a monotherapy in 17 patients (30.9%) and as a rescue treatment in 38 patients (69.1%) (Fig. 1). There was no significant difference in age (74 (39–88) vs. 64 (25–89), *p* = 0.18), percentage of male patients (58.9% vs. 58.2%, *p* = 0.88), or percentage of treatment with anticoagulants (8.0% vs. 5.5%, *p* = 0.75) or antithrombotic agents (28.6% vs. 16.4%, *p* = 0.09) or both (9.8% vs. 9.1%, *p* = 0.550) between the conventional and UI-EWD groups. Moreover, the two groups were similar in terms of laboratory findings. The UI-EWD groups was significantly higher in patients who were expected to have high comorbidity with a Charlson Comorbidity Index of 6 or high (41.4% vs. 70.9%, *p* = 0.048) (Table 1). The median follow-up duration was not significantly different between the conventional and UI-EWD groups (131 (7–668) vs. 47 (7–797), *p* = 0.674).

**Table 1:** Baseline clinical characteristics of study subjects

Variables	Conventional therapy (n = 112)	UI-EWD therapy (n = 55)	*P*-value
Age (year)^§^	74 (39–88)	64 (25–89)	0.181
Sex (male)^§^	66 (58.9)	32 (58.2)	0.875
*Medication, n (%)*			
Anticoagulant	9 (8.0)	3 (5.5)	0.753^+^
Antiplatelet agent	32 (28.6)	9 (16.4)	0.085
Anticoagulant and antiplatelet agent	11 (9.8)	5 (9.1)	0.549^+^
White blood cell (1000/μL) ^§^	6580 (2100–20,570)	7270 (2290–36,410)	0.337
Hb (g/dL)^§^	10.0 (12.0–4.9)	12.4 (6.4–16.0)	0.369
Platelet count (1000/μL)^§^	208 (55–654)	222 (49–371)	0.550
PTs (INR)^§^	1.03 (0.84–5.18)	1.05 (0.84–18.40)	0.320
aPTT (s)^§^	36.5 (24.2–79.2)	36.3 (26.2–79.2)	0.457
BUN (mg/dL)^§^	16.4 (5.5–139.8)	15.5 (6.5–95.3)	0.372
Cr (mg/dL)^§^	0.87 (0.29–8.43)	0.83 (0.29–7.28)	0.502
CCI ≥ 6, n (%)	46 (41.4)	39 (70.9)	0.048
Follow-up duration (day)^§^	131 (7–668)	47 (7–797)	0.674

UGI, Upper gastrointestinal; LGI, Lower gastrointestinal; Hb, Hemoglobin; PTs, Prothrombin time; aPTT, Activated partial thromboplastin time; BUN, Blood urea nitrogen; Cr, Creatinine; CCI, Charlson Comorbidity index

^§^Median (range)

^+^Fisher’s exact test

*P-values were calculated using the *t*-test or Fisher calculated using the of the conventional therapy group and the UI-EWD therapy group

### Bleeding characteristics of the conventional group and the UI-EWD therapy group

The bleeding characteristics of the study subjects are shown in Table 2. The UI-EWD group was significantly more applied to the hepatic flexure (7.3% vs. 0%, *p* = 0.01) and slightly more applied to the sigmoid colon (18.2% vs. 11.6%, *p* = 0.247).

**Table 2:** Bleeding characteristics of the study subjects

Variables	Conventional therapy (n = 112)	UI-EWD therapy (n = 55)	*P*-value
*Location of bleeding, n (%)*			
Cecum	5 (4.5)	2 (3.6)	0.625
Ascending colon	18 (16.1)	5 (9.1)	0.219
Hepatic flexure	0 (0.0)	4 (7.3)	0.011^+^
Transverse colon	12 (10.7)	4 (7.3)	0.478
Descending colon	5 (4.5)	1 (1.8)	0.388
Sigmoid colon	13 (11.6)	10 (18.2)	0.247
Rectum	59 (52.7)	29 (52.7)	0.830
*Cause of bleeding, n (%)*			
Diverticular bleeding	13 (11.6)	4 (7.3)	0.204
Radiation proctitis	13 (11.6)	1 (1.8)	0.037^+^
Angiodysplasia	9 (8.0)	1 (1.8)	0.168^+^
Ulcer bleeding	34 (30.4)	18 (32.7)	0.756
Post procedure bleeding	31 (27.7)	25 (45.5)	0.147^+^
Tumor bleeding	12 (10.7)	6 (10.9)	0.970
*Size of bleeding site, n (%)*			
< 1 cm	71 (63.4)	15 (27.3)	< 0.001
1–4 cm	32 (28.6)	26 (47.3)	0.022
> 4 cm	9 (8.0)	14 (25.5)	0.002
*Forrest classification, n (%)*			
Ia	2 (1.8)	5 (9.1)	0.040^+^
Ib	112 (94.6)	50 (90.1)	0.001^+^
*Treatment modality, n (%)*			
Coagrasper	38 (33.0)	22 (40.0)	0.397
APC	21 (18.8)	5 (9.1)	0.106
Hemoclipping	81 (53.6)	21 (38.2)	< 0.001
EVL	4 (3.6)	0 (0.0)	0.156
UI-EWD only	0 (0.0)	17 (30.9)	

^+^Fisher exact test

*P-values were calculated using the *t*-test or Fisher calculated using the of the conventional therapy group and the UI-EWD therapy group

Differences in the cause of bleeding between the two groups were evaluated. Radiation proctitis was significantly more common in the conventional therapy group (11.6% vs. 1.8%, *p* = 0.037) due to the higher use of argon plasma coagulation.

Bleeding sites > 1 cm (including more than 4 cm) were significantly more common in the UI-EWD therapy group than in the conventional therapy group (1–4 cm: 47.3% vs. 28.6%, *p* = 0.02, > 4 cm: 25.5% vs. 8.0%, *p* < 0.001).

According to the initial status of bleeding, for Forrest Ib, diffuse oozing bleeding, hemostasis with conventional therapy seemed sufficient (94.6% vs. 90.1%, *p* = 0.001); otherwise, additional UI-EWD treatment was significantly more applied to Forrest Ia, active spurting bleeding (9.1% vs. 1.8%, *p* = 0.04).

There was no significant difference in treatment modalities between the two groups, with the exception of hemoclipping, which was significantly more common in the conventional therapy group (53.6% vs. 39.3%, *p* < 0.001).

### Comparison of clinical outcomes after bleeding control between the conventional group and the UI-EWD therapy group

**Fig. 4:**
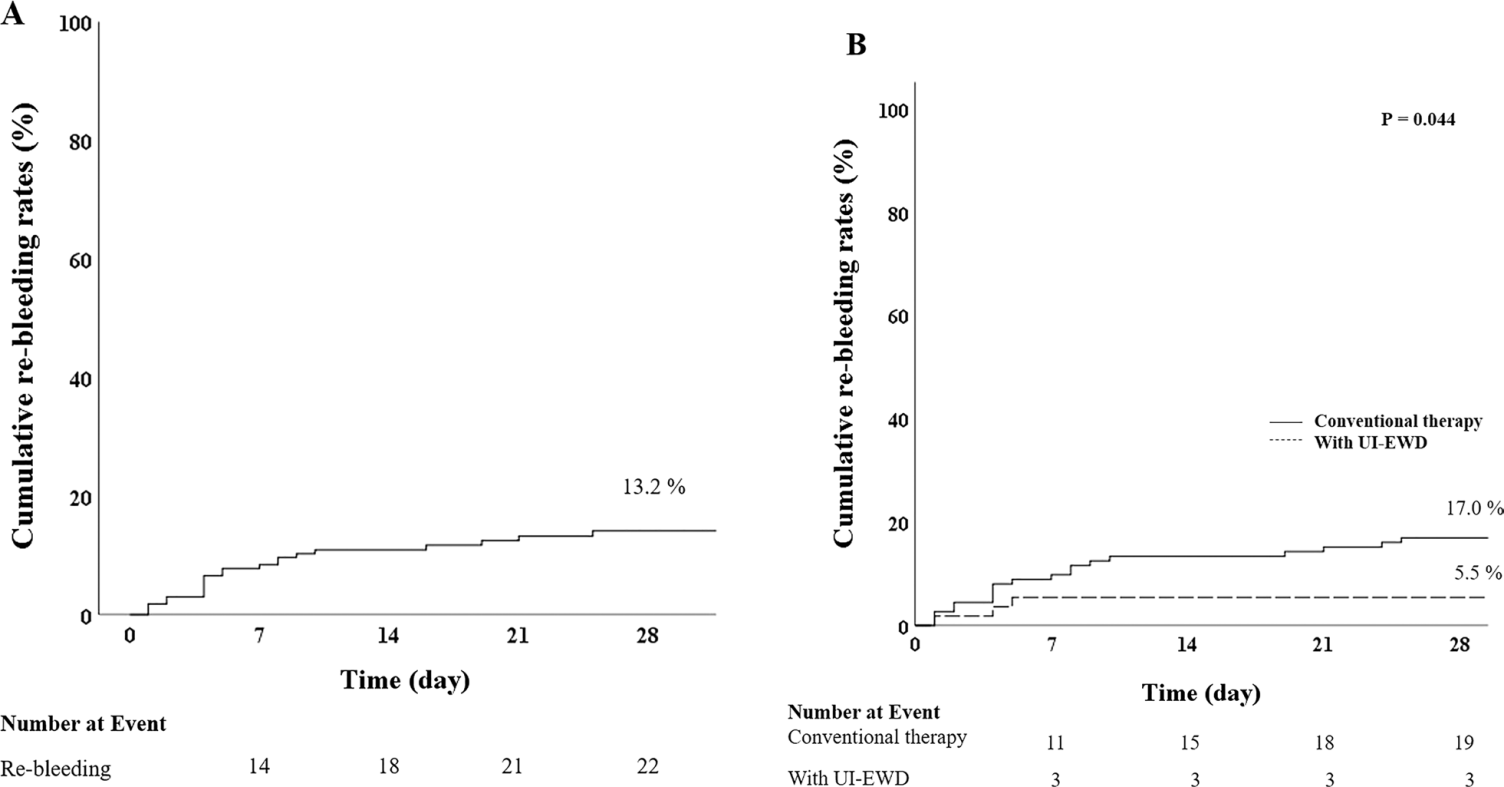
Cumulative re-bleeding rates in all enrolled patients (**A**) and according to treatment modality (**B**)

All patients in both groups showed successful immediate hemostasis. However, the cumulative rebleeding rate within 28 days was significantly lower in the UI-EWD therapy group (5.5% vs. 17%, *p* = 0.04) (Table 3 and Fig. 4). The main difference in rebleeding rate between the two groups was determined within 7 days (12.5% vs. 5.5%, *p* = 0.12), and the re-bleeding rate was lower after 7 days in both groups. Moreover, the UI-EWD group did not show re-bleeding after 7 days. The median time to rebleeding was within 7 days, 5 days (1–25) in the conventional therapy group, and 4 days (1–5) in the UI-EWD therapy group with no significant difference.

**Table 3:** Clinical outcomes of bleeding control

Variables	Conventional therapy (n = 112)	UI-EWD therapy (n = 55)	*P*-value
Success of immediate hemostasis, n (%)	112 (100%)	55 (100%)	
*Cumulative re-bleeding, n (%)*			
At 7 days	14 (12.5)	3 (5.5)	0.157
At 14 days	16 (14.3)	3 (5.5)	0.091
At 28 days	19 (17.0)	3 (5.5)	0.039
Time to re-bleeding (days)^§^	5 (1–25)	4 (1–5)	
*Adverse event, n (%)*			
Colonic obstruction	0 (0.0)	0 (0.0)	-
Perforation	1 (0.9)	0 (0.0)	-
Infection	1 (0.9)	0 (0.0)	0.448^+^
Embolization	0 (0.0)	0 (0.0)	-

^§^Median (range)

^+^Fisher’s exact test

*P-values were calculated using the *t*-test or Fisher calculated using the of the conventional therapy group and the UI-EWD therapy group

There were no reported adverse events associated with UI-EWD application; otherwise, one patient in the conventional therapy group showed perforation with infection after hemostasis by hemoclipping (Table 3). In terms of mortality, there were no case of patient’s death during the follow-up period in both group.

### Bleeding characteristics and clinical outcomes of the UI-EWD monotherapy group

Of the 55 patients in the UI-EWD therapy group, 17 (30.9%) received UI-EWD as monotherapy. We performed a subgroup analysis of bleeding characteristics and clinical outcomes according to UI-EWD monotherapy (Table 4). All of the monotherapy groups had initial diffuse oozing bleeding (Forrest Ib), most of the lesions were > 1 cm (16/17, 94.1%), with a different indication of cancer (5/17, 29.4%), post ESD or EMR bleeding (10/17, 58.8%), and ulcer (2/17, 11.8%). There was no rebleeding within 28 days of UI-EWD monotherapy. Moreover, no UI-EWD-associated adverse events, such as perforation or colonic obstruction, were recorded.

**Table 4:** Bleeding characteristics and clinical outcome of patients in the UI-EWD monotherapy group

Variables	UI-EWD monotherapy (n = 17)
*Location of bleeding, n (%)*	
Hepatic flexure	2 (11.8)
Transverse colon	3 (17.6)
Sigmoid colon	4 (23.5)
Rectum	8 (47.6)
*Cause of bleeding, n (%)*	
Ulcer bleeding	2 (11.8)
Post procedure bleeding	10 (58.8)
Tumor bleeding	5 (29.4)
*Size of bleeding site, n (%)*	
< 1 cm	1 (5.9)
1–4 cm	10 (58.8)
> 4 cm	6 (35.3)
*Forrest classification, n (%)*	
Ia	0 (0.0)
Ib	17 (100.0)
*Cumulative re-bleeding, n (%)*	
At 7 days	0 (0.0)
At 14 days	0 (0.0)
At 28 days	0 (0.0)
Time to re-bleeding (days)^§^	–
*Adverse event, n (%)*	
Colonic obstruction	0 (0.0)
Perforation	0 (0.0)
Infection	0 (0.0)
Embolization	0 (0.0)

^§^Median (range)

^+^Fisher’s exact test

*P-values were calculated using the *t*-test or Fisher calculated using the of the conventional therapy group and the UI-EWD therapy group

## Discussion

UI-EWD powder is a newly developed topical hemostatic agent. Use of this powder in refractory upper GI bleeding, non-variceal bleeding, and diffuse tumor bleeding has been described as safe and efficient for the prevention of rebleeding [18–21]. In the present study, we showed another promising result of UI-EWD on LGIB in that it prevented cumulative re-bleeding rate down to 5.5%, showing a greater advantage in sites that are difficult to access by endoscopy or those that are larger with diffuse oozing bleeding.

The rebleeding rate in the UI-EWD group was 5.5%, which is a considerable improvement compared to the rate of 12.8% observed with previously reported hemostatic powders in LGIB [16]. In addition to the common advantages of hemostatic powders, including easier accessibility and greater applicability to larger and diffuse oozing bleeding lesions [24, 25], differences in the composition of hemostatic powders might affect the hemostatic efficiency. Although there has been no head-to-head comparison, UI-EWD contains aldehyde dextran as a main component mixed with ε-poly amino acid at a 4:1 ratio, while other commercially available powders are mainly composed of polysaccharides [20]. These differences in UI-EWD components are thought to lower the rebleeding rate by increasing the adhesion power from the Schiff base reaction through the cross-linking of amine and aldehyde dextran groups when they come into contact with moisture. In previous reports, the UI-EWD hydrogel was still present at 70.2% of the sprayed bleeding sites by second-look endoscopy at 24 h [20].

The characteristic of hemostatic powder working as locally, a shorter hemostatic effect is inevitable under the conditions of GI tract movement and food or feces passing. In addition, in clinical settings, it has been reported that the rebleeding rate further increases with time despite a high rate of successful initial hemostasis as a result of using hemostatic powder in upper and lower GI bleeding [26]. Moreover, there was a high rate of rebleeding after application of Hemospray (33.7%), with similar rates for both esophagogastroscopies and colonoscopies (35.6% vs. 23.1%). Most reported rebleeding occurred within 7 days (86.2%) [27]. However, in our report, the UI-EWD group did not show delayed rebleeding between 7 and 28 days after the initial successful hemostasis in LGIB. In our study, rebleeding was reported in three cases within 7 days; however, no rebleeding was reported after 7 days.

The medical records of the three patients with rebleeding were reviewed. The first patient showed rebleeding 1 day after endoscopic submucosal dissection of a 3-cm Yamada type III polyp diagnosed with rectal cancer. Owing to the taller (as opposed to wide) shape, there is a high possibility that a thick feeding vessel was hidden by the remnant submucosal injection, which can cause active arterial bleeding. The second patient showed rebleeding 4 days after initial hemostasis of a rectal ulcer with underlying liver cirrhosis and a low platelet count of 20,000/μL. These high bleeding tendencies could not escape from the rebleeding. The last patient showed rebleeding 5 days after initial hemostasis of a transverse colon ulcer and took dual antiplatelet agents due to a history of cardiac stent insertion. In addition to the hemostasis failure of initial clipping on diffuse oozing bleeding ulcers, clipping itself might promote bleeding by GI movement. Although there were only three rebleeding cases, patients with a higher bleeding tendency or those who bled from the rectum. Further, a well-designed prospective study of various conditions that increase rebleeding is needed.

In the UI-EWD monotherapy group (Table 4), most of the applied cases were post-procedure or tumor bleeding (15/17, 88.2%), with sizes of bleeding site > 1 cm (16/17, 94.1%). In these cases, additional endoscopic hemostasis was hesitated because of the higher risk of delayed perforation. Despite these difficult circumstances, a single application of UI-EWD seemed to be sufficient to prevent rebleeding and allow initial hemostasis. Moreover, there was no reported perforation or colonic obstruction due to the single use of UI-EWD. This is meaningful in that applying mono-treatment of hemostatic powder is less traumatic and may be a reliable option for active oozing bleeding with larger size, especially for post procedure or tumor bleeding in LGIB. This is compatible with the findings of our previous report on the effectiveness of UI-EWD for upper GI tumor bleeding [18].

In the comparison of bleeding characteristics between the conventional therapy group and the UI-EWD therapy group, UI-EWD was more commonly applied to the hepatic flexure. This is known as one of the most difficult areas to access or to fix the endoscopic position due to its sharp angulation, and more frequently has larger sizes > 1 cm, even ≥ 4 cm. Even under these adverse conditions, the UI-EWD group showed a significantly lower cumulative rebleeding rate within 28 days (5.5%) than the conventional therapy group (17%).

Considering the adverse events of visceral perforation and splenic infarction reported previously when using Hemospray [28], we also evaluated UI-EWD-related adverse effects, including embolism, intestinal obstruction, and allergic reaction, due to the characteristics of the powder. However, there were no reported cases in the UI-EWD group compared to perforation or infection reported by coagulation treatment in the conventional therapy group. In case of perforation and embolism by Hemospray, it was considered due to the high pressure when injecting powder, which induces a patient’s risk of abdominal pain after applying Hemosrapy [16]. As a result of direct comparison of pressure between UI-EWD and Hemospray, UI-EWD was significantly lower at 7 psi than at 37 psi. These low injection pressures may have contributed to the safety of UI-EWD.

Our study has some limitations. First, it was a retrospective analysis at a single-center hospital, which may not be generalizable. Second, the patients in whom UI-EWD was applied were not randomly distributed and the decision to use is left to the discretion of the physicians, which could present a selection bias. Despite these limitations, bleeding sites in the UI-EWD therapy group were more difficult to endoscopically approach, such as hepatic flexure or sigmoid colon, and were more common in the longer diameter group. To obtain further information on the practical performance of UI-EWD in LGIB and to evaluate the feasibility and efficacy of LGIB, a well-designed, large-scale, prospective study is needed.

In conclusion, this is the first study to demonstrate the effectiveness and safety of UI-EWD in the treatment of LGIB. Application of UI-EWD resulted in a high rate of immediate hemostasis (100%) and a significant reduction in re-bleeding in patients with LGIB whose condition is not well controlled by conventional treatment (17% vs. 5%, *p* = 0.044). Based on our results, we suggest that the use of UI-EWD for hemostasis in LGIB is sufficiently effective and safe.

## Data Availability

The datasets used and analyzed during the current study are not publicly available due to keeping privacy of patients but are available from the corresponding author on reasonable request.
